# Early-Life Nutrition Interventions Improved Growth Performance and Intestinal Health via the Gut Microbiota in Piglets

**DOI:** 10.3389/fnut.2021.783688

**Published:** 2022-01-03

**Authors:** Chengzeng Luo, Bing Xia, Ruqing Zhong, Dan Shen, Jiaheng Li, Liang Chen, Hongfu Zhang

**Affiliations:** ^1^State Key Laboratory of Animal Nutrition, Institute of Animal Science, Chinese Academy of Agricultural Sciences, Beijing, China; ^2^College of Animal Science, Xinjiang Agricultural University, Urumqi, China; ^3^College of Animal Science and Technology, Northwest A&F University, Xianyang, China

**Keywords:** early-life nutrition interventions, growth performance, intestinal barrier, inflammatory cytokines, short-chain fatty acids (SCFAs), gut microbiota

## Abstract

Intestinal infections in piglets are the main causes of morbidity before and after weaning. Studies have not explored approaches for combining pre-weaning and post-weaning nutritional strategies to sustain optimal gut health. The current study thus sought to explore the effects of early-life nutrition interventions through administration of synthetic milk on growth performance and gut health in piglets from 3 to 30 days of age. Twelve sows were randomly allocated to control group (**CON**) and early-life nutrition interventions group (**ENI**). Piglets were fed with the same creep diet from 7 days of age *ad libitum*. Piglets in the ENI group were provided with additional synthetic milk from Day 3 to Day 30. The results showed that early-life nutrition interventions improved growth performance, liver weight, spleen weight, and reduced diarrhea rate of piglets after weaning (*P* < 0.05). Early-life nutrition interventions significantly upregulated expression of ZO-1, *Occludin, Claudin4, GALNT1, B3GNT6*, and *MUC2* in colonic mucosa at mRNA level (*P* < 0.05). Early-life nutrition interventions reduced activity of alkaline phosphatase (AKP) in serum and the content of lipopolysaccharides (LPS) in plasma (*P* < 0.05). The number of goblet cells and crypt depth of colon of piglets was significantly higher in piglets in the ENI group relative to that of piglets in the CON group (*P* < 0.05). The relative mRNA expression levels of *MCP-1, TNF-*α, *IL-1*β, and *IL-8*, and the protein expression levels of TNF-α, IL-6, and IL-8 in colonic mucosa of piglets in the ENI group were lower compared with those of piglets in the CON group (*P* < 0.05). Relative abundance of *Lactobacillus* in colonic chyme and mucosa of piglets in the ENI group was significantly higher relative to that of piglets in the CON group (*P* < 0.05). Correlation analysis indicated that abundance of *Lactobacillus* was positively correlated with the relative mRNA expression levels of *ZO-1, Claudin4*, and *GALNT1*, and it was negatively correlated with the level of *MCP-1* in colonic chyme and mucosa. In summary, the findings of this study showed that early-life nutrition interventions improved growth performance, colonic barrier, and reduced inflammation in the colon by modulating composition of gut microbiota in piglets. Early-life nutrition intervention through supplemental synthetic milk is a feasible measure to improve the health and reduce the number of deaths of piglets.

## Introduction

Stress is a major cause of death in piglets. Approximately 10 million piglets die each year due to stress all over the world (1). Piglets are subjected to a number of stressors, such as an abrupt separation from the sow, transportation and handling stress, social hierarchy stress, comingling with pigs from other litters, and a different physical environment (room, building, farm, and water supply) during pre- and post-weaning periods (2). Changes in food are also an important source of stress. Creep feed is often provided to piglets in swine industries to improve post-weaning growth performance of piglets and improve acclimatization of piglets to solid feed before weaning. The process of dietary gradual transition from sow milk to solid feed is an important stressor for piglets owing to the significant differences between creep feed and sow milk (3). Piglets are subjected to bear risk of viruses and pathogens from solid feed, resulting in intestinal diseases, especially diarrhea (3). Moreover, dietary transition often leads to disorder in digestive function, which in turn causes low growth performance (4). Notably, low-birth-weight piglets may die easier in the transition period (5).

Gut microbiome plays a fundamentally significant role in the health of the host (6). Increase in the abundance of beneficial intestinal microflora can reduce occurrence of stress caused by sudden dietary transition by reducing the frequency of diarrhea and intestinal inflammation in the host (7). Studies report that nutritional interventions can modulate the abundance of intestinal microbes, and reduce the risk of gastrointestinal infections (3). Early-life nutrition intervention in piglets has been widely explored in recent years. Several studies have explored effects of oral supplementation, including prebiotics and functional amino acids, for neonatal piglets and the findings show that these oral supplementations increase abundance and colonization of beneficial bacteria, and improve gut health of the host (8). For example, dietary supplementation with 1% amino acid blend improved intestinal functions, and reduced incidence of diarrhea in piglets (9). Moreover, yeast glycoprotein supplementation increases relative abundance of *Lactobacillus* in the colon of piglets (10). In addition, synthetic milk modulates mucosal immunology and abundance of microbiota in neonatal piglets (11). Notably, synthetic milk reduces the abundance of *Escherichia* and diarrhea frequency in piglets after weaning (7). Therefore, early-life nutritional intervention has significant potential in reducing the risk of gastrointestinal infections in piglets. Furthermore, it promotes growth performance in piglets (12). Tan et al. (13) reported that pigs with high-feed efficiency exhibit large numbers of beneficial bacteria in their gut.

Moreover, synthetic milk plays a nutritional role in piglets. Milk production in sows may not satisfy the demand of piglets when litters comprise more piglets relative to the number of productive sow teats. Therefore, some piglets face severe starvation stress during lactation which ultimately result in poor growth performance or even death (14). Piglets should thus be supplied with additional nutritional interventions to improve growth performance. A previous study reported that administration of synthetic milk increased body weight of weaning piglets by 18% (8). In addition, synthetic milk significantly reduces pre-weaning mortality (15). However, only a limited number of studies have explored effects of nutritional interventions on intestinal microbes and growth performance. Moreover, previous studies reported a wide variation in the timing of administration of the supplements, the category of piglets, the type of supplements, and supplementation dosage (3). Therefore, further studies should explore pre-weaning and post-weaning nutritional strategies that sustain optimal growth performance and intestinal health throughout the weaning process for piglets. The present study thus proposes the hypothesis: early-life nutrition interventions improve growth performance and intestinal health by modulating gut microbiota abundance in piglets. This study thus sought to explore the effects of early-life nutrition interventions on growth performance and gut health of piglets, through determination of body weight, diarrhea rate, weight of visceral organs, effect on intestinal barrier, expression levels of inflammatory cytokines in the colon, profiles of microbial metabolites, and colonic microbiota composition.

## Materials and Methods

### Ethics Statement

All procedures in the current study including animal experiments and sample collection were approved by the Experimental Animal Welfare and Ethical Committee of the Institute of Animal Science, Chinese Academy of Agricultural Sciences (No. IAS2020-104).

### Experimental Design

A total of 12 healthy sows (3–4 years old, 5th−6th parity) obtained from a commercial farm (Henan, China) along with their litters were assigned to two groups: the control group (**CON**) and the early-life nutrition intervention group (**ENI**). Each group comprised a total of 6 sows, and 11 piglets were selected from each litter. The piglets among the two groups had similar initial body weight (initial BW = 1.73 ± 0.03 kg). All sows with suckling piglets were separately housed in closed farrowing pens and provided with similar commercial diets and water *ad libitum*. The environment of the pigpens was kept clean, ventilated, and regularly disinfected.

**Figure 1:**
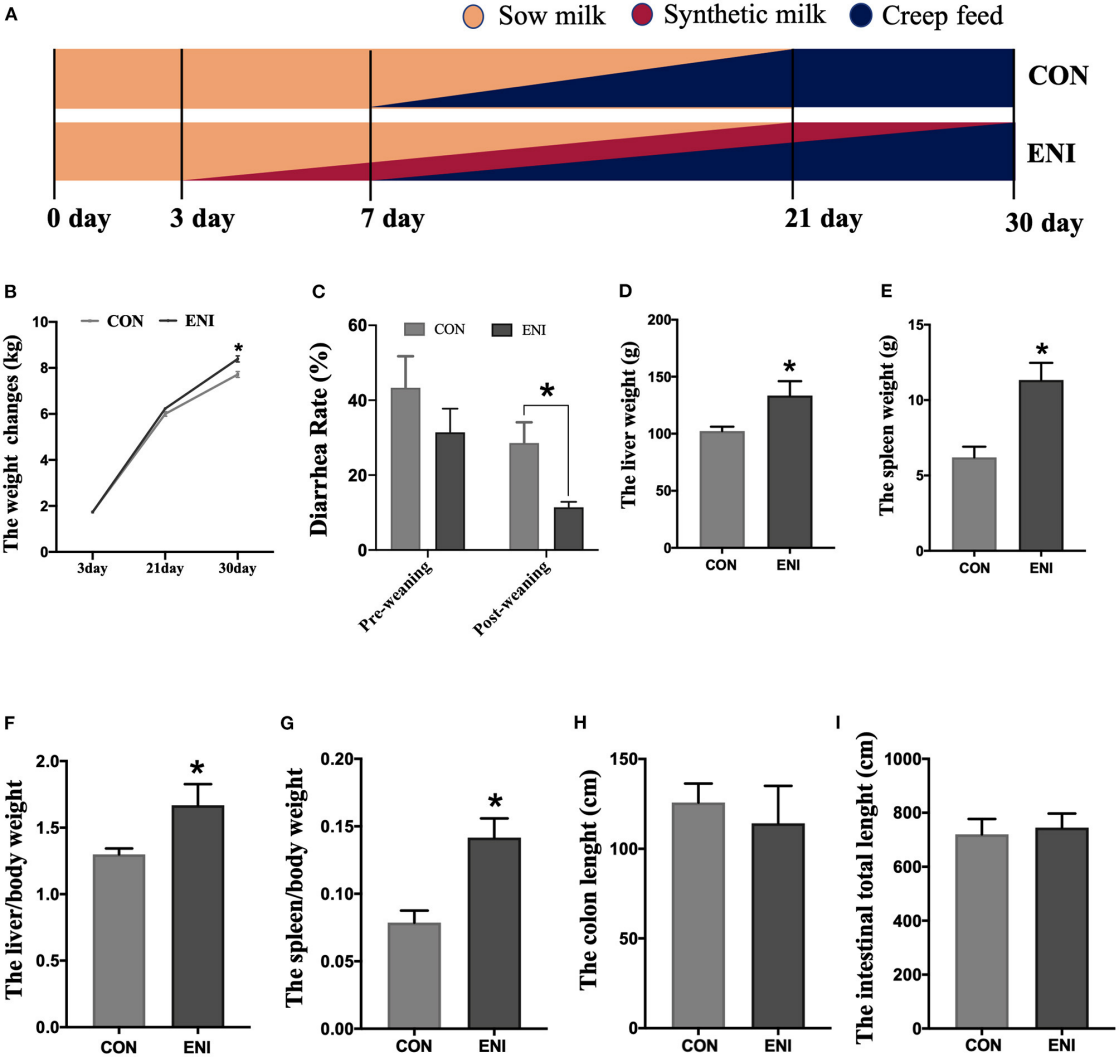
Effects of early-life nutrition interventions on growth performance and visceral organs in piglets. CON, control group, ENI, early-life nutrition interventions group. **(A)** The feeding mode diagram. **(B)** The weight changes of piglets at 3, 21, and 30 days of age in each group. **(C)** The diarrhea rate of pre-weaning and post-weaning of piglets. **(D)** The liver weight of piglets in each group. **(E)** The spleen weight of piglets in each group. **(F)** The relative weights of liver to living body weight. **(G)** The relative weights of spleen to living body weight. **(H)** The length of colon in each group. **(I)** The length of whole intestine. Data are expressed as mean ± SEM (*n* = 4). The independent-samples *t*-test was used to compare data between two groups. *P < 0.05.

All piglets were fed with the same standard creep diet (Table 1) from 7 to 30 days of age. Newborn piglets in the CON group were only fed with the standard creep diet. Newborn piglets in the ENI group were provided with additional synthetic milk, a supplementary for breast milk from Day 3 to Day 30 of age. All piglets were weaned on Day 21. Piglets in the ENI group were allowed free access to synthetic milk before 22 days of age, whereas the amount of synthetic milk was reduced from 22 to 30 days. Piglets were fed with synthetic milk through feeders. The synthetic milk and feeders were purchased from Libaowei Nutrition Technology Co., Ltd (Guangdong, China). Synthetic milk was treated with ultrahigh temperature methods. Details on the feeding mode and the nutritional constituents of the synthetic milk are presented in Figure 1A and Table 1, respectively. The living weight of all piglets was recorded on Day 3, 21 and Day 30 of age. Incidence of diarrhea of piglets was recorded every day during the experimental period and the diarrhea rate was calculated as follows: Diarrhea rate (%) = (the number of piglets with diarrhea × diarrhea days) / (total number of piglets × total observational days) × 100 (16).

**Table 1:** Composition and nutrient levels of diets^a^.

**Ingredient composition, %**	**Diet**
	**CON**
Corn	57.00
Full-fat soybean	6.00
Soybean meal	20.00
Fish meal	5.00
Dried whey	5.00
Soybean oil	1.00
CaHPO4	0.50
NaCl	0.30
Limestone	0.51
Choline chloride	0.09
Lysine HCl	0.40
Met	0.10
Thr	0.10
Glucose	1.50
Suger	1.50
Premix^b^	1.00
Nutrient levels, %	
DE (MJ/kg)	14.44
CP	20.68
Lys	1.18
Met	0.37
Total Ca	0.70
Total P	0.55

a *E, digestible energy; CP, crude protein*.

b *The premix provided the following per kg of diets: VA: 18,000 IU; VD3: 4500 IU; VE 22.5 mg; VK3: 4.5 mg; VB1: 4.32 mg; VB2: 12 mg; VB6: 4.86 mg; VB12: 0.03 mg; nicotinamide: 41.58 mg; calcium pantothenate: 33.12 mg; folic acid: 1.764 mg; biotim: 0.48 mg; Cu: 20 mg; Fe: 140 mg; Zn: 140 mg; Mn: 40 mg; I: 0.5 mg; Se: 0.3 mg*. *The synthetic milk was added to the ENI group based on the standard creep diets. The proximate composition of the synthetic milk (% dry matter): crude protein, 19.50%; crude lipid, 17.50%; crude ash, 6.00%; crude fiber, 0.00%; calcium, 0.60%; phosphorus, 0.60%; potassium, 1.40%; sodium, 0.50%; Lys, 1.90%; Met+Cys, 0.95%; Thr, 1.00%; Trp, 0.32%*.

### Sample Collection

Four piglets randomly selected from each group were weighed at the end of the experiment (Day 30 of the pigs' age). Blood samples of 5 ml were collected from the anterior vena cava of the piglets. Blood samples were coagulated for 30 min, then serum was obtained by centrifugation at 3,000 g for 10 min at ambient temperature. Furthermore, plasma was collected using heparin as an anticoagulant, and the plasma sample was centrifuged for 10 min at 3,000 g. The piglets were euthanized under anesthesia after collection of blood samples. The length of the colon and the whole intestine was determined. Further, the weight of livers and spleen was determined. Samples of colonic chyme and mucosa were collected, immediately transferred to liquid nitrogen, and stored at −80°C for subsequent analysis. Colon tissue samples were fixed in Carnoy's solution.

### Genes Expression Analysis

Total RNA was extracted using TRIzol reagent (Ambion, UT) from colonic mucosa samples. RNA integrity was by electrophoresis using 1.0% agarose gel. Nano Drop^®^ ND-1000 spectrophotometer (Nano-Drop Technologies, Wilmington, DE) was used to determine quality of RNA. cDNA was synthesized from RNA using a reverse transcription kit. cDNA samples were stored at −20°C for quantitative real-time PCR (qRT-PCR). All primers were designed by the National Center for Biotechnology Information (NCBI). Primer sequences are presented in Table 2. Primers were purchased from Sangon Biotech (Shanghai, China). qRT-PCR assays were performed using TB Green Premix Ex Taq (TaKaRa, Kusatsu, Japan) at a final volume of 20 μL containing 10 μl of 2 × Top Green qPCR SuperMix (TransStart^®^, Beijing, China), and 7.8 μl of ddH_2_O, 0.4 μl of each primer, 1 μl of diluted cDNA, and 0.4 μl of Passive Reference Dye (50 ×). x*ZO-1, Claudin4, Occludin, TNF-*α, *IL-1*β, *MCP-1, IL-8, GALNT1, B3GNT6, MUC2* mRNA expression levels were explored in colonic mucosa. Expression levels of the target genes were normalized to the housekeeping genes β*-actin* and *GAPDH* as endogenous controls. qRT-PCR reaction was programmed as follows: 94°C for 30 s, and followed by 40 cycles for 5 s at 94°C, 30 s at 60°C and 10 s at 72°C. Relative expression levels of all genes were determined using the 2^−ΔΔCT^ method (17).

**Table 2:** The nucleotide sequences of primer.

**Target gene**	**Forward sequence (5′- 3′)**	**Reverse sequence (5′- 3′)**
ZO-1	CTCCAGGCCCTTACCTTTCG	GGGGTAGGGGTCCTTCCTAT
Claudin4	CAACTGCGTGGATGATGAGA	CCAGGGGATTGTAGAAGTCG
Occludin	CAGGTGCACCCTCCAGATTG	TATGTCGTTGCTGGGTGCAT
TNF-α	TAAGGGCTGCCTTGGTTCAG	AGAGGTTCAGCGATGTAGCG
IL-1β	ATTCAGGGACCCTACCCTCTC	CTTCTCCACTGCCACGATGA
MCP1	AAACGGAGACTTGGGCACAT	GCAAGGACCCTTCCGTCATC
IL-8	TACGCATTCCACACCTTTC	GGCAGACCTCTTTTCCATT
GALNT1	GAGCCCAGTGATGGATGGAT	GGGAACACTTGGCCTTTCAG
B3GNT6	CTGGAGTGTTGTCCAGCCAT	AGCTAAGGAGCAGCGTCAAG
MUC2	CGCATGGATGGCTGTTTCTG	ATTGCTCGCAGTTGTTGGTG
GAPDH	GGGCATGAACCATGAGAAGT	GGGCATGAACCATGAGAAGT
β-actin	GCGTAGCATTTGCTGCATGA	GCGTGTGTGTAACTAGGGGT

### AKP, LPS, and ELISA

AKP activity in serum was determined using commercial reagent kits (Nanjing Jiancheng Bioengineering Institute, Nanjing, China). LPS level in plasma was determined using the commercially available Tachypleus amebocyte lysate kit (Chinese Horseshoe Crab Reagent Manufactory Co., Ltd, Xiamen, China) following the quantitative Chromogenic Limulus Amebocyte Lysate assay method.

Protein expression levels of IL-8, IL-6, and TNF-α proteins in the colonic mucosa were determined using ELISA assay kits (Invitrogen, Carlsbad, CA) according to the manufacturer's protocol. A microplate reader (BioTek-ELx808, BioTek Instruments, Inc., Texas) was used to read the protein bands.

### Tissue Sample and Intestinal Morphology Analysis

Colon tissue was immersed and fixed with Carnoy's solution for 24 h. Colon samples were then removed from formalin and embedded in paraffin. Subsequently, the paraffin blocks were sectioned to obtain 5 μm thick sections using a semi-automatic microtome (LONGSHOU, China). Sections were stained with hematoxylin and eosin (H&E) stain (18), and viewed under an optical microscope. The crypt depth was determined following a method described by Wang et al. (19).

Further, 5 μm thick sections were obtained as described above and analyzed using the PAS-AB kit (Beijing Solarbio Technology Co., Ltd, Beijing, China) instructions. Sections were viewed under an optical microscope, and the amount of goblet cells was determined using a method reported by Cantero-Recasens et al. (20).

### Analysis of SCFAs Levels

Approximately 0.1 g of colonic chyme and mucosa samples were separately obtained from each sample. The samples were suspended in 1 mL of ddH_2_O in 1.5-mL screw capped vials for analysis of concentration of SCFAs. Concentrations of SCFAs were determined by gas chromatography as described by Wu et al. (21).

### DNA Extraction, 16S RRNA Gene Amplification, Sequencing and Analysis

Approximately 0.5–1 g of colonic chyme and colonic mucosa samples were separately obtained from each sample, and microbial community genomic DNA was extracted using E.Z.N.A.^®^ soil DNA Kit (D5625-02, Omega Bio-Tek Inc., Norcross, GA) according to the manufacturer's instructions. Genomic DNA samples were stored at −80°C for subsequent analysis. Purity and DNA concentration were determined through 1% agarose gel electrophoresis and NanoDrop2000 spectrophotometer (Thermo Fisher Scientific, Waltham, MA), respectively. V3-V4 regions of bacterial 16S rRNA gene were amplified using the following primer set: 338F (5'-ACTCCTACGGGAGGCAGCAG-3') and 806R (5'-GGACTACHVGGGTWTCTAAT-3'). The reaction system was comprised of 4 μl of 5 × FastPfu Buffer, 2 μl of 2.5 mM dNTPs, 0.8 μl of each primer (5 μM), 0.4 μl of FastPfu polymerase and 10 μl of DNA template. The reactions were performed using GeneAmp^®^ 9700 thermal cycler (Applied Biosystems, Foster City, CA). Amplification process was as follows: denaturation for 3 min at 95°C followed by 27 cycles of 95°C for 30 s, 55°C for 30 s, 72°C for 45 s, and a final extension of 10 min at 72°C. The amplified fragments were analyzed by electrophoresis on a 2% agarose gel. The products were then purified with AxyPrep DNA Gel Extraction Kit (Axygen Bioscience, CA) according to the manufacturer's instructions. Raw microbial sequence data were analyzed and processed at the Majorbio Bio-Pharm Technology Co. Ltd. (Shanghai, China). Sequences were analyzed and assigned to operational taxonomic units (OTUs; 97% identity). The alpha-diversity whose coverage is based on the Chao and Shannon index within each sample was determined by QIIME tool (Version 174 1.7.0) (22). Beta diversity was evaluated by computing the unweighted Unifrac distance and visualized using principal coordinates analysis (PCoA) plots. LDA effect size (LEfSe) was used to explore biomarkers that exhibited statistical differences. The datasets presented in this study were submitted to the NCBI Sequence Read Archive (SRA) database. The name of the repository and accession number is [PRJNA779481].

### Statistical Analysis

Growth performance, organs weight, intestinal length and morphology, colonic permeability, mRNA expression, and concentrations of SCFAs data were subjected to analysis of variance using SPSS (23.0) software. Student's *t*-test was used to determine differences between two groups. The results were presented as means ± SEM. The relationships among the intestinal barrier, inflammatory cytokines, and bacterial species were explored using Pearson's correlation analysis and a correlation matrix was generated. Figures were generated using GraphPad 8.0 software. * was used to indicate a statistically significant difference (*P* < 0.05), and ** indicated a highly significant difference (*P* < 0.01). The *P* values ranging from 0.05 to 0.1 were also recorded.

## Results

### Body Weight, Diarrhea Rate, and Organ Development in Piglets

The results showed that the body weight of piglets in both groups gradually increased from Day 3 to Day 30. The body weight of piglets in the ENI group was significantly higher relative to that of piglets in the CON group at the age of 30 (*P* < 0.05) (Figure 1B). Diarrhea rate of piglets in the ENI group was lower compared with the rate of piglets in the CON group, and the difference was statistically significant at the post-weaning stage (*P* < 0.05) (Figure 1C). Piglets in the ENI group had higher liver and spleen weight and higher relative weights of liver and spleen to living body weight compared with those of piglets in the control group (*P* < 0.05) (Figures 1D–G). The colon of piglets in the CON group was longer relative to that of piglets in the ENI group, however, the differences were not statistically significant (Figures 1H,I).

### Intestinal Morphology and Intestinal Barrier of Colonic Mucosa in Piglets

**Figure 2:**
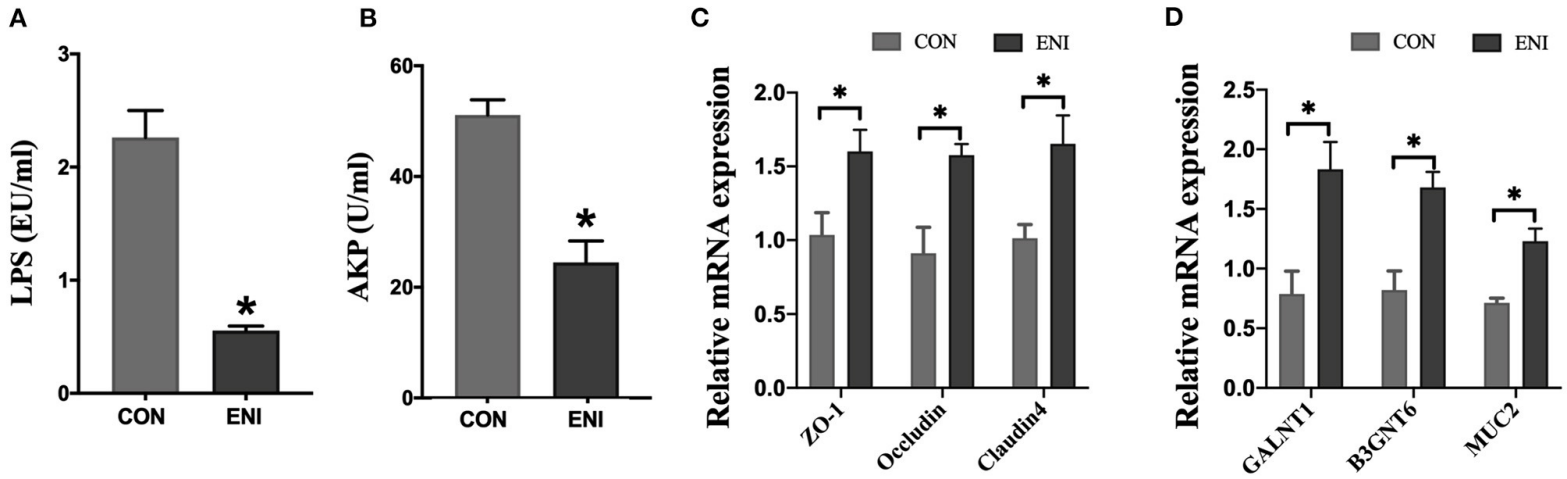
Effects of early-life nutrition interventions on the intestinal barrier of the colon in piglets. CON, control group, ENI, early-life nutrition interventions group. **(A)** The content of LPS in plasma. **(B)** The activity of AKP in serum. **(C)** The mRNA expression levels of ZO-1, Occludin, and Claudin4 in the colon. **(D)** The mRNA expression levels of GALNT1, B3GNT6, and MUC2 in the colon. Data are expressed as mean ± SEM (*n* = 4). The independent-samples *t*-test was used to compare data between two groups. *P < 0.05.

The results showed that the activity of AKP in serum and the level of LPS in plasma of piglets in the ENI group were significantly lower compared with those of piglets in the CON group (*P* < 0.05) (Figures 2A,B). The findings indicated that early-life nutrition intervention significantly upregulated expression of *ZO-1, Occludin, Claudin4, GALNT1, B3GNT6*, and *MUC2* genes in colonic mucosa at the mRNA level (Figures 2C,D).

**Figure 3:**
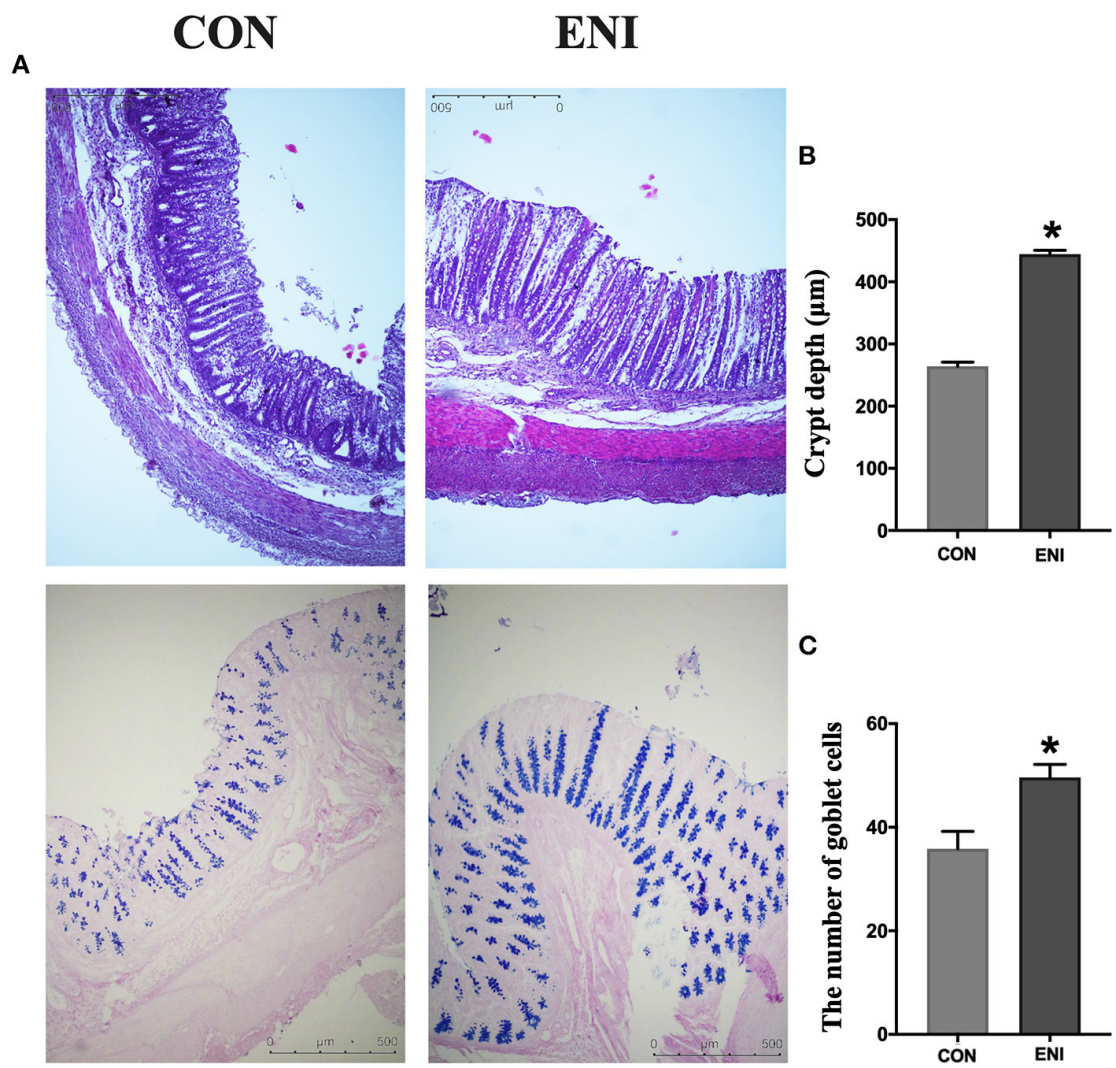
Effects of early-life nutrition interventions on the morphology of the colon and the number of goblet cells. CON, control group, ENI, early-life nutrition interventions group. **(A)**. HE and PAS staining of colon tissue. **(B)** The crypt depth of the colon. **(C)** The number of goblet cells of the colon. Data are expressed as mean ± SEM (*n* = 4). The independent-samples t-test was used to compare data between two groups. *P < 0.05.

Analysis of intestinal morphology did not show any noticeable pathologic changes in the two groups; however, the number of goblet cells and crypt depth of piglets was significantly higher in the ENI group compared with that of the CON group (*P* < 0.05) (Figures 3A–C).

### Intestinal Inflammation in Colonic Mucosa of Piglets

**Figure 4:**
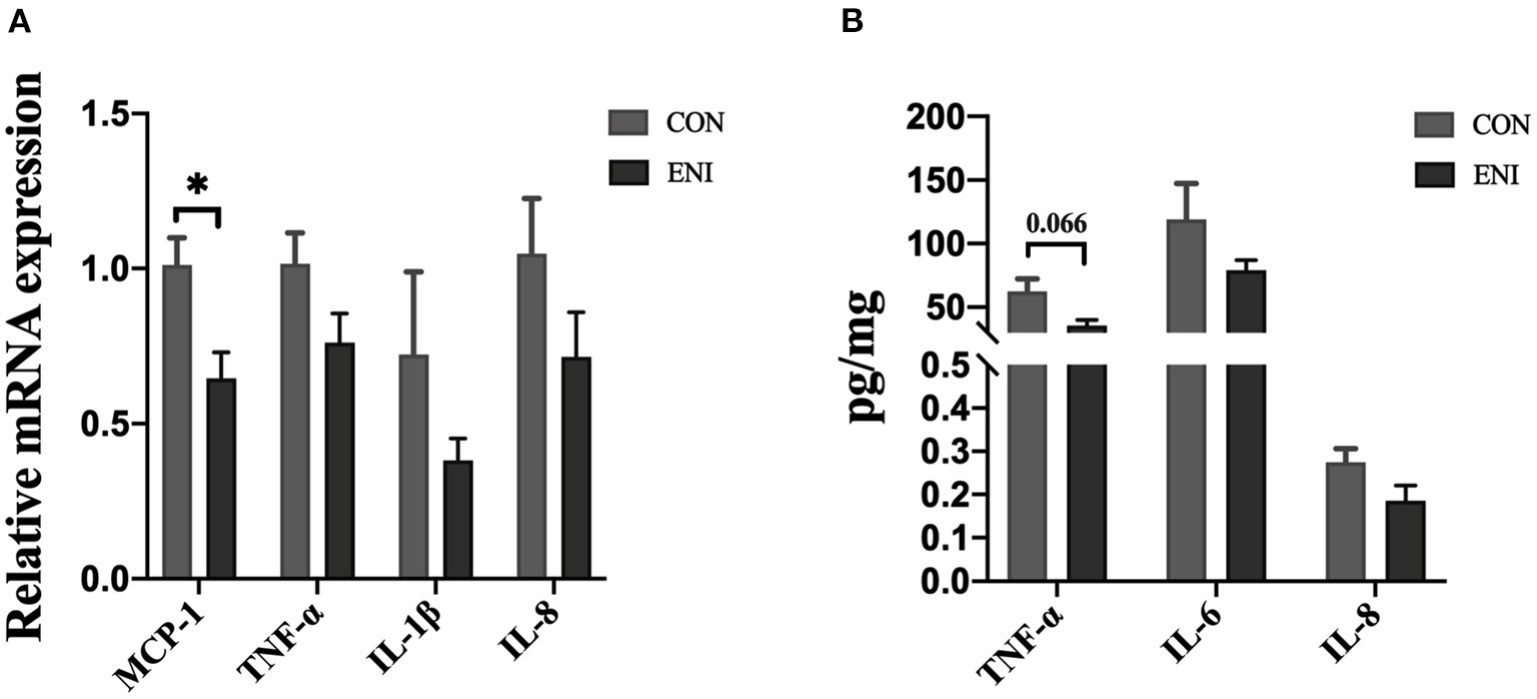
Effects of early-life nutrition interventions on inflammation in the piglet colon. CON, control group, ENI, early-life nutrition interventions group. **(A)** The mRNA expression levels of MCP-1, TNF-α, IL-1β, and IL-8 in colon. **(B)** The protein expression levels of TNF-α, IL-6, and IL-8 in piglets. Data are expressed as mean ± SEM (*n* = 4). The independent-samples *t*-test was used to compare data between two groups. *P < 0.05.

Relative mRNA expression levels of MCP-1, TNF-α, IL-1β, and IL-8 in the ENI group were lower relative to those in the CON group, however, only the difference in relative mRNA expression level of MCP-1 was statistically significant (*P* < 0.05) (Figure 4A). Protein expression levels of inflammatory cytokines (TNF-α (*P* = 0.066), IL-6, and IL-8) in the colonic mucosa showed a similar trend as the relative mRNA expression levels of inflammatory cytokines (Figure 4B).

### Concentrations of SCFAs in Colonic Mucosa and Chyme

**Figure 5:**
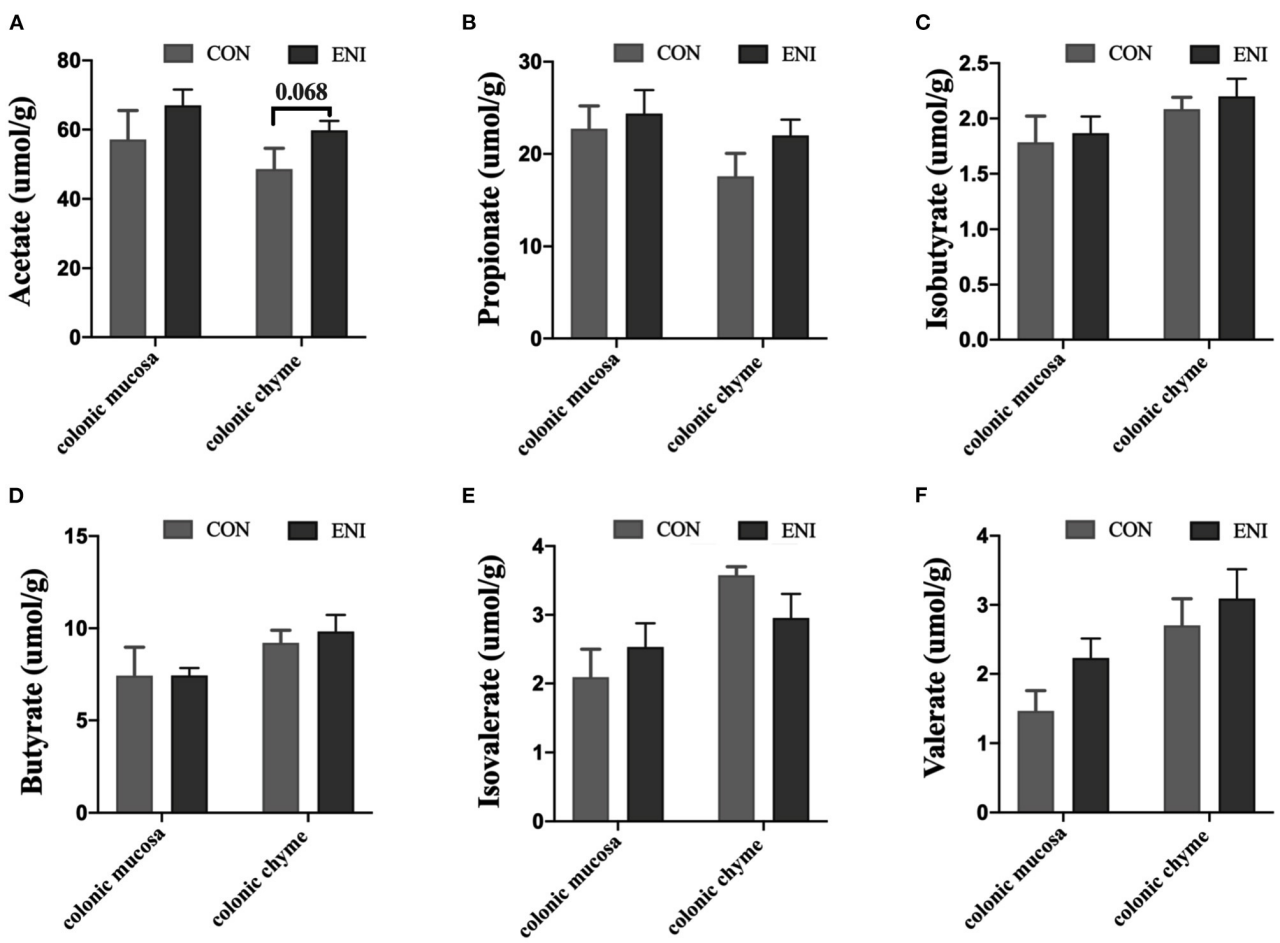
Effects of early-life nutrition interventions on concentrations of SCFAs in the colonic mucosa and colonic chyme. CON, control group, ENI, early-life nutrition interventions group. **(A)** Acetate acid. **(B)** Propionic acid. **(C)** Isobutyric acid. **(D)** Butyric acid. **(E)** Isovaleric acid. **(F)** Valeric acid. Data are expressed as mean ± SEM (*n* = 4). The independent-samples *t*-test was used to compare data between two groups. *P < 0.05.

Concentrations of acetic acid, propionic acid, isobutyric acid, butyric acid, and valeric acid in colonic mucosa and colonic chyme of piglets in the ENI group were higher relative to those of piglets in the CON group (Figures 5A–D,F), however, the differences were not statistically significant. Concentration of isovaleric acid in colonic chyme of piglets in the ENI group was lower compared with that of piglets in the CON group, however, the difference was not statistically significant (Figure 5E).

### Composition of Gut Microbes in Colonic Chyme and Mucosa Samples From Piglets

**Figure 6:**
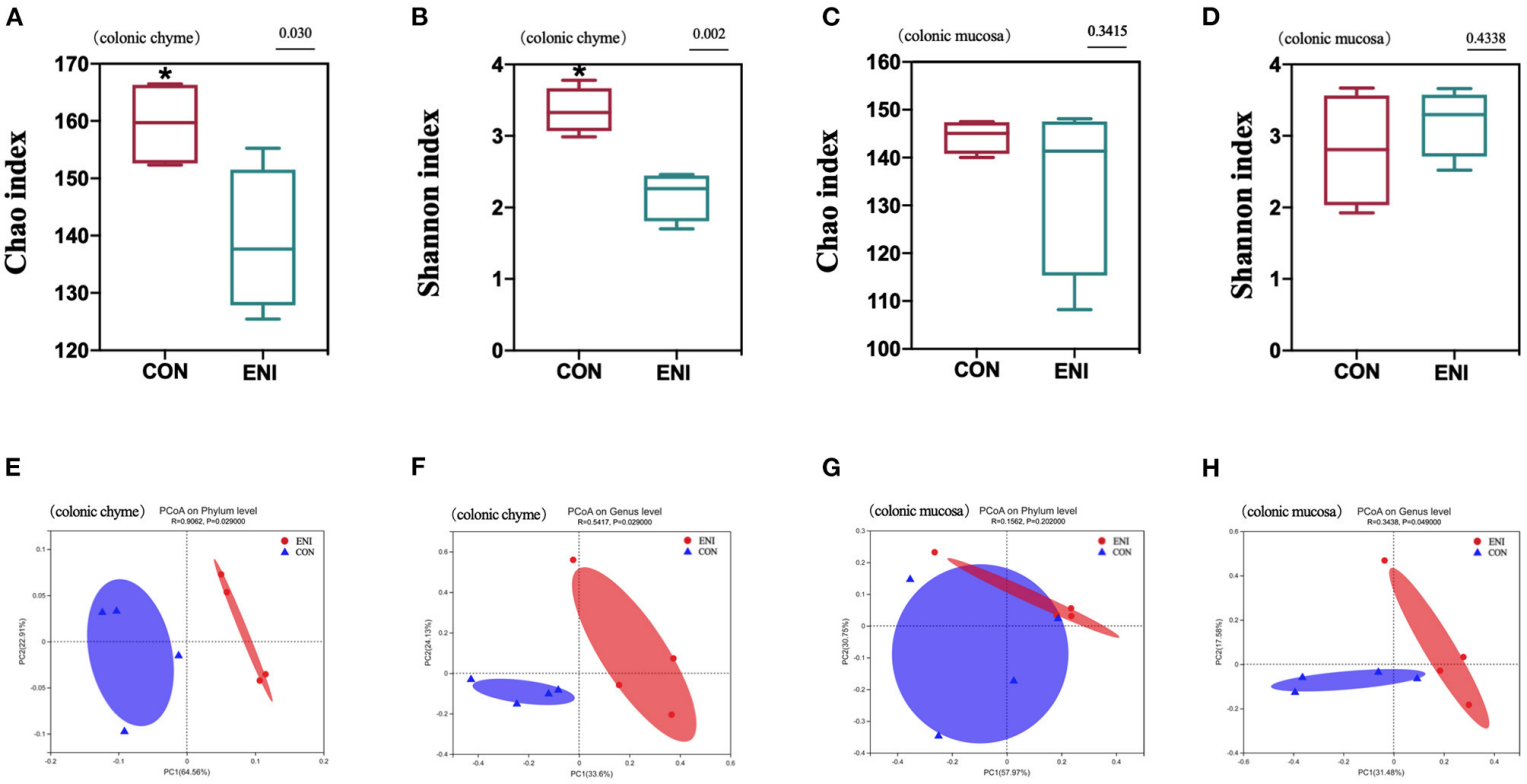
The analysis of gut microbiota diversity in the colonic chyme and colonic mucosa of piglets. CON, control group, ENI, early-life nutrition interventions group. **(A)** The Chao index of microbiota in colonic chyme. **(B)** The Shannon index of microbiota in colonic chyme. **(C)** The Chao index of microbiota in colonic mucosa. **(D)** The Shannon index of microbiota in colonic mucosa. **(E)** PCoA analysis of microbiota in colonic chyme at the phylum level. **(F)** PCoA analysis of microbiota in colonic chyme at the genus level. **(G)** PCoA analysis of microbiota in colonic mucosa at the phylum level. **(H)** PCoA analysis of microbiota in colonic mucosa at the genus level. Data are expressed as mean ± SEM (*n* = 4). The independent-samples *t*-test was used to compare data between two groups. *P < 0.05.

Fresh colonic chyme and colonic mucosa were obtained from piglets, and 16 s rRNA gene sequencing analysis was performed to explore the effects of early-life nutrition interventions on the structures and composition of the gut microbiota. The findings showed no significant difference in Chao index and Shannon index in colonic mucosa between the two group (Figures 6C,D). However, Chao index and Shannon index in colonic chyme of piglets in the CON group were higher compared with those of piglets in the ENI group (*P* < 0.05) (Figures 6A,B). PCoA analysis showed significant differences in phylum and genus composition between ENI and CON groups (Figures 6E–H).

**Figure 7:**
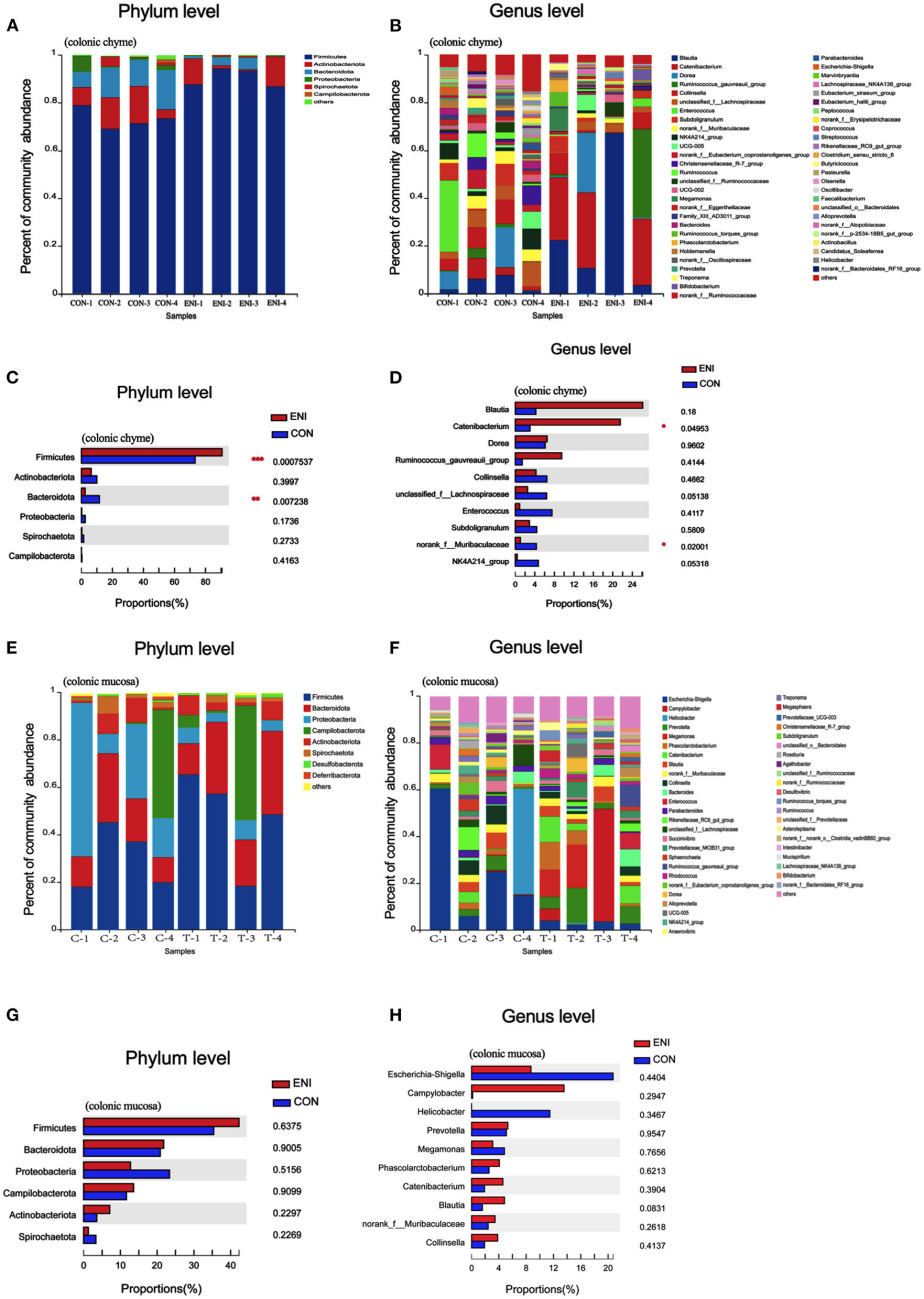
Gut microbiota community composition in the colonic chyme and colonic mucosa of piglets. CON, control group, ENI, early-life nutrition interventions group. **(A)** The relative abundances of microbiota in colonic chyme at the phylum level. **(B)** The relative abundances of microbiota in colonic chyme at the genus level. **(C)** The top 6 bacteria in colonic chyme at the phylum level statistical comparison of the relative abundances. **(D)** The top 10 bacteria in colonic chyme at the genus level statistical comparison of the relative abundances. **(E)** The relative abundances of microbiota in colonic mucosa at the phylum level. **(F)** The relative abundances of microbiota in colonic mucosa at the genus level. **(G)** The top 6 bacteria in colonic mucosa at the phylum level statistical comparison of the relative abundances. **(H)** The top 10 bacteria in colonic mucosa at the genus level statistical comparison of the relative abundances. Data are expressed as mean ± SEM (*n* = 4). The independent-samples *t*-test was used to compare data between two groups. *P < 0.05.

Microbial community composition at the phylum and genus level of the two groups is presented in Figures 7A,B,E,F. The results showed that colonic chyme samples comprised 6 major phyla including *Firmicutes, Actinobacteria, Bacteroidota, Proteobacteria, Spirochaetota*, and *Campilobacterota* (Figure 7A). The findings showed that colonic mucosa samples mainly comprised *Desulfobacterota, Deferribacterota, Firmicutes, Actinobacteria, Bacteroidota, Proteobacteria, Spirochaetota*, and *Campilobacterota* at the phylum level (Figure 7E). The proportion of *Firmicutes* in colonic chyme was higher in the ENI group compared with that in the CON group, whereas the proportion of *Bacteroidota* in colonic chyme was higher in the CON group relative to that in the ENI group (*P* < 0.05) (Figure 7C). Notably, the proportion of the top 6 bacteria in colonic mucosa was not significantly different between the two groups (Figure 7G).

**Figure 8:**
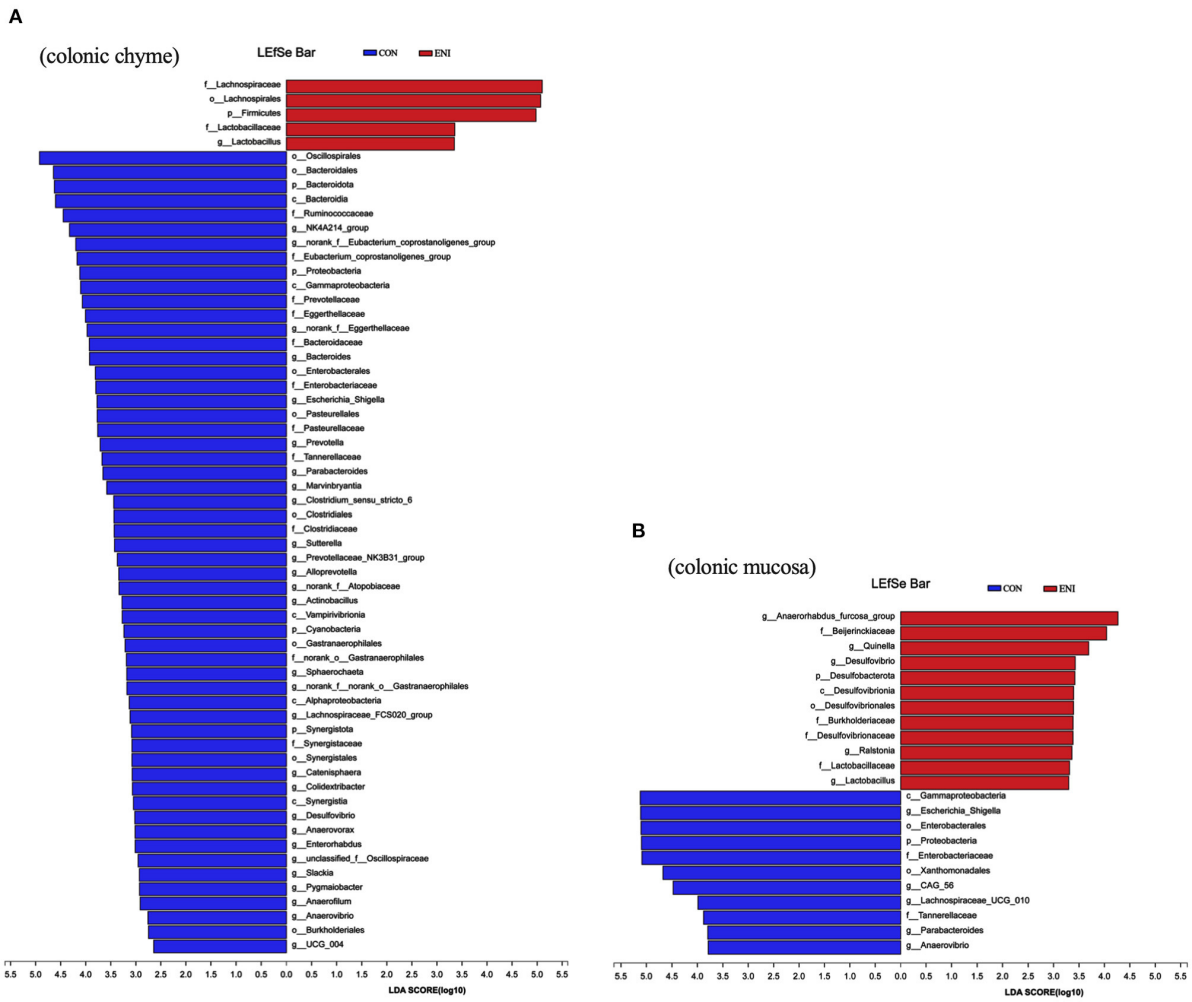
The differentially abundant taxa among the two groups by LEfSe analysis. CON: control group, ENI: early-life nutrition interventions group. **(A)** LEfSe analysis of microbiota in colonic chyme. **(B)** LEfSe analysis of microbiota in colonic mucosa.

Composition of the gut microbiota in the colonic chyme and colonic mucosa was further analyzed at the genus level. The proportion of *Catenibacterium* in colonic chyme was significantly higher in the ENI group (*P* < 0.05), whereas the proportion of *norank-f-Muribaculaceae* in colonic chyme was lower in the ENI group compared with that of the CON group (*P* < 0.05) (Figure 7D) The proportion of the top 10 bacteria in colonic mucosa was not significantly different between the two groups (Figure 7H). LEfSe analysis was performed and presented as LDA score ≥2.0 to further explore the differences in microbiota composition between the two groups. The results showed that 61 biomarkers (blue bar) were enriched in colonic chyme in the CON group compared with the levels in the ENI group. Notably, LEfSe analysis showed that only 5 biomarkers (red bar) were enriched in colonic chyme of the ENI group, including *Lachnospiraceae, Lachnospirales, Firmicutes, Lactobacillaceae*, and *Lactobacillus* (Figure 8A). Analysis of colonic mucosa showed a total of 23 biomarkers in the CON and ENI groups. The results showed that piglets in the ENI group had a higher composition of microbiota compared with that in the CON group (Figure 8B). Notably, *Lactobacillus* was enriched in colonic mucosa and chyme.

### Relationship Among Intestinal Microbiota, Intestinal Barrier and Immune-Related Indexes

**Figure 9:**
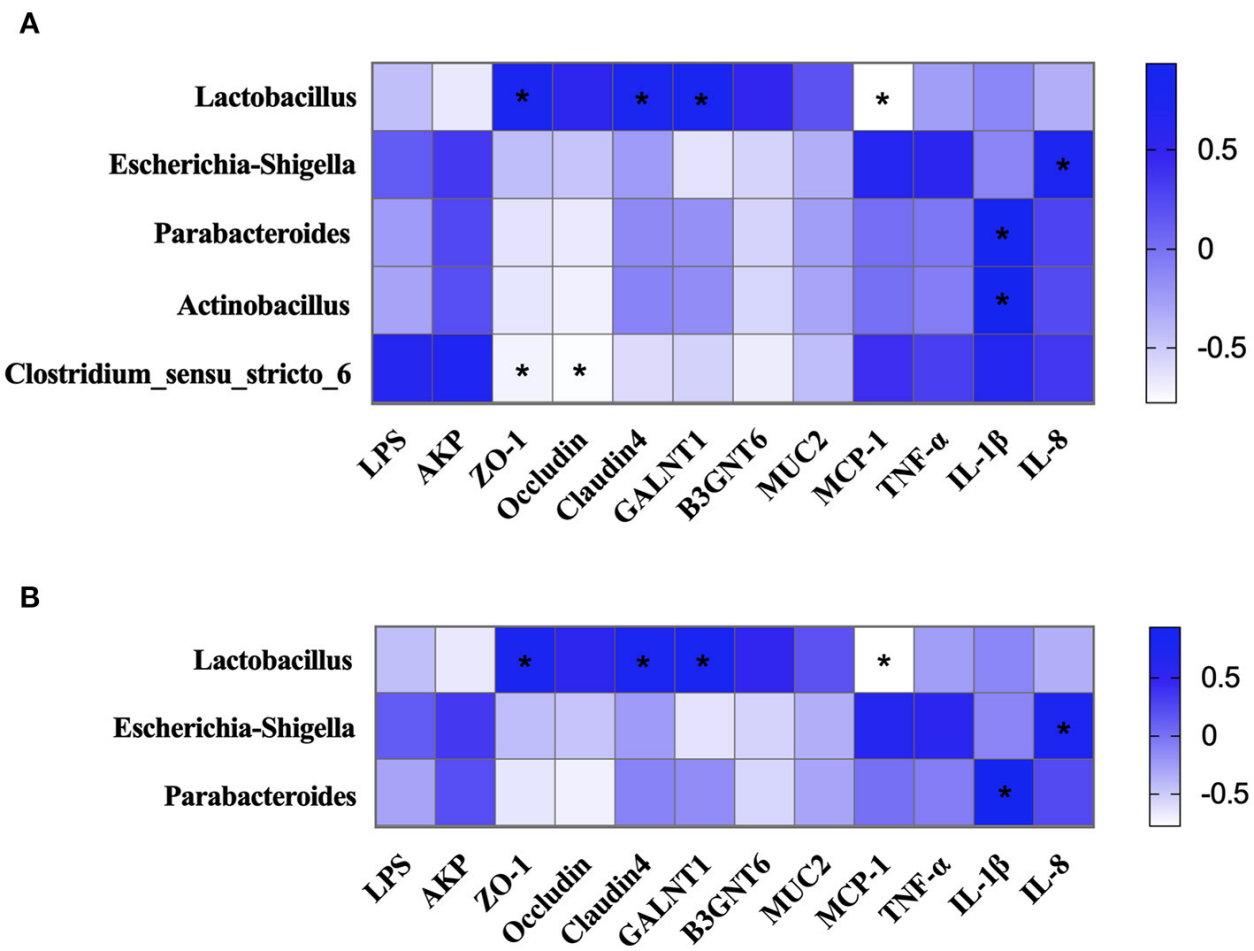
The correlation of intestinal microbiota, intestinal barrier, and immune-related indexes. **(A)** The correlation of intestinal microbiota, intestinal barrier, and immune-related indexes in colonic chyme. **(B)** The correlation of intestinal microbiota, intestinal barrier, and immune-related indexes in colonic mucosa. *P < 0.05.

The potential association among intestinal microbiota, intestinal barrier, and immune-related indexes in colonic chyme and mucosa was explored. The results showed that the proportion of *Lactobacillus* in colonic chyme was negatively correlated with the relative mRNA expression level of *MCP-1 and* was positively correlated with the relative mRNA expression levels of *ZO-1, Claudin4*, and *GALNT1* (Figure 9A). The proportion of *Escherichia-Shigella* was positively correlated with the relative mRNA expression level of *IL-8*. Moreover, proportions of *Parabacteroides* and *Actinobacillus* were positively correlated with the relative mRNA expression level of *IL-1*β, whereas the proportion of *Clostridium-sensu-stricto-6* was negatively correlated with the relative mRNA expression levels of *ZO-1* and *Occludin*.

**Figure 10:**
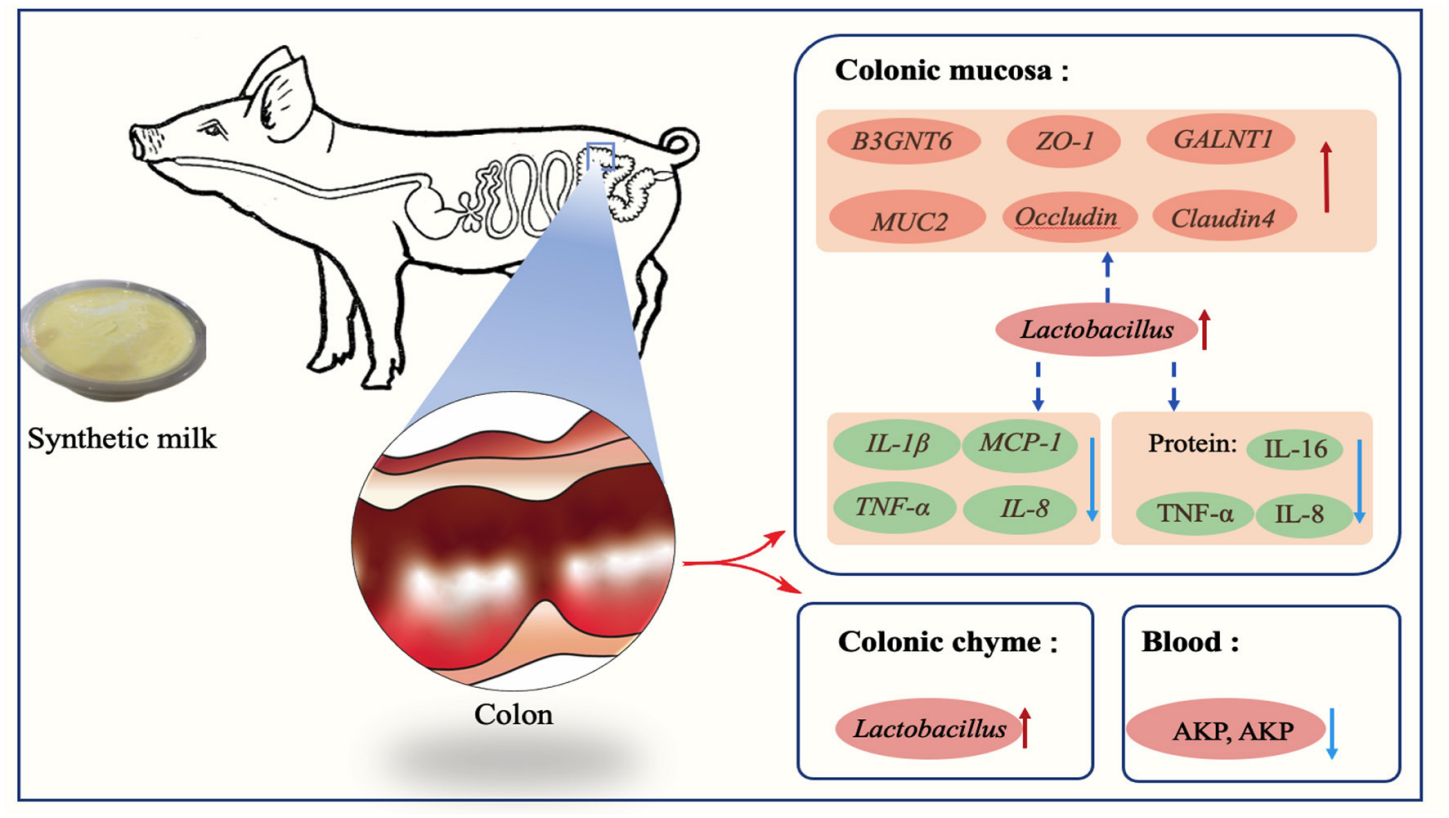
Early-life nutrition interventions improved growth performance and intestinal health via the gut microbiota in piglets: a possible mechanism.

Correlation analysis of the intestinal microbiota, intestinal barrier, and immune-related indexes in colonic mucosa showed similar results to those of colonic chyme (Figure 9B). The results showed that the proportion of *Lactobacillus* was negatively correlated with the relative mRNA expression level of *MCP-1*, and was positively correlated with the relative mRNA expression levels of *ZO-1, Claudin4*, and *GALNT1*. The proportion of *Escherichia-Shigella* was positively correlated with the relative mRNA expression levels of *IL-8*. The proportion of *Parabacteroides* was positively correlated with the relative mRNA expression level of *IL-1*β (Figure 10).

## Discussion

Previous studies report that dietary changes can alter the composition of gut microbes in suckling piglets and these changes can be adjusted by early-life nutrition interventions (3, 23). For example, Sugiharto et al. (11) reported that provision of synthetic milk to suckling piglets improved the gut microbiome composition, as well as increased concentration of SCFAs in the colon. Similar findings were observed in the present study. The proportion of *Firmicutes* and *Bacteroidetes* was significantly different between the ENI and CON groups. Studies report that change in microbiota composition is significantly associated with diet composition. A diet rich in protein and fat increases the ratio of *Firmicutes* to *Bacteroides* in the intestines of animals or humans (24). The synthetic milk used in the current study contained high crude protein and crude fat content, which may be an important reason for the increase in *Firmicutes* proportion in the intestine and a decrease in *Bacteroides* proportion. Most previous studies mainly focused on microbiota composition in chyme and fecal samples, and only a few studies explored the composition of mucosa-associated microbiota (25). Changes in mucosa-associated microbiota may have significant effects on host growth and development (25). Therefore, microbiome composition in chyme and mucosa was explored and a significant difference was observed between the two sites, both at phylum and genus levels. This disparity can be attributed to different substrate availability, and rapid decrease in oxygen gradient from the outer mucosal layer to the lumen (26, 27). Although the microbiome composition was different in chyme and mucosa samples in this study, *Lactobacillus* was the dominant genus in the ENI group. *Lactobacillus* genus is commonly found in food and intestinal tracts and is a typical probiotic with significant beneficial effects on gut health. *Lactobacillus* effectively inhibits colonization of harmful bacteria in the intestine (28). A high proportion of *Lactobacillus* can be attributed to administration of early-life nutritional interventions, resulting in low proportions of harmful bacteria, such as *Escherichia-Shigella, Actinobacillus, Clostridium-sensu-stricto-6, Pasteurellaceae*, and *Parabacteroides*.

The period from birth to 30 days old is a transition period during which the piglet diet is changed from sow milk to solid foods, and this period represents a delicate moment for piglets (29, 30). Weaned piglets are more likely to suffer from body weight loss, appetite slough, and digestive function disorder during this period (31). Several studies have been conducted to explore the changes that occur during this period as they affect production in the pig industry (32). Studies report that supplementation of additional formula milk improves growth performance, and reduces incidence of pre-weaning and post-weaning diarrhea of piglets (7), and the findings show that gut microbiota play a key role in these changes. The findings of the present study showed that early-life nutrition interventions improve body weight gain and reduce diarrhea incidence at pre-weaning and post-weaning periods of piglets. Early-life nutrition interventions significantly increased the ratio of liver to body weight and ratio of spleen to body weight. The ratio of liver to body weight is widely used as a parameter for general assessment of liver size or regrowth. The spleen is a major hematopoietic tissue, and the increased ratio of spleen to body weight is correlated with the hematopoietic function of the spleen (33). This finding indicates that the condition of the piglets improved after administration of early-life nutrition interventions. *Lactobacillus* was enriched in colonic mucosa and chyme of piglets. These findings indicate that early-life nutrition interventions supported growth of beneficial bacteria in the gut before and after weaning thus reducing the risk of gastrointestinal infections, and improved body weight of piglets (3). Moreover, the proportion of *Firmicutes* was increased, whereas the proportion of *Bacteroidetes* was significantly decreased in the ENI group relative to that of the CON group. Previous studies report that the ratio of *Firmicutes* to *Bacteroidetes* is an indicator of obesity (34). *Firmicutes* are more efficient in absorbing energy compared with *Bacteroidetes*, thus they promote more efficient absorption of calories resulting in weight gain (35). This finding partially explains the observation that piglets in the ENI group exhibited better growth performance compared with the CON group. In addition, studies report that milk substitutions improve feed intake of piglets (36), which is often used as a measure of growth performance (37). Therefore, increase in the weight of piglets in the ENI group can be attributed to improved feed intake induced by the early-life nutrition interventions.

The intestine is one of the largest organs in organisms and plays a role in defending other organs against harmful agents, and plays key roles in digestion and absorption of dietary nutrients in the body (38). Nutrients are received from the outside to the intestine to support the life of animals, and various harmful pathogens and toxins may attack the host through the intestine (39). The intestinal barrier plays an important role in life activities and prevents these harmful substances from penetrating the intestinal tissue (40). The results in the current study showed that the relative mRNA expression levels of tight junction proteins, barrier defense-related proteins, and key glycosyltransferases were higher in piglets in the ENI group relative to the levels in piglets in the CON group. High expression levels of these markers implied that synthetic milk improves the gut barrier function, and maintains the intestine at a relatively healthy level (41). Previous studies used milk as a substitute for sow milk and the findings showed that it improved intestinal function and upregulated expression of tight junction proteins. For example, Jin et al. (42) reported that milk supplementation changes mRNA expression levels of *ZO-1, Occludin*, and *Claudin-1*. The synthetic milk may potentially harbor specific growth factors which improved expression of tight junction proteins (42). Moreover, *Lactobacillus* may have upregulated gene expression of tight junction proteins (43). Previous studies report that *Lactobacillus* plays a key role in positively protecting the gut barrier (44). Pearson correlation analysis was conducted to explore the relationships among the abundances of major gut flora with the expression of tight junction proteins. Abundance of *Lactobacillus* was positively correlated with expression levels of tight junction proteins explored in the present study. The *MUC2* gene plays an important role in intestinal barrier protection and is produced and secreted by intestinal goblet cells (45). Several factors regulate the secretory processes of *MUC2* including cytokines, toxins, microbiota-metabolites, and microbes such as *Lactobacillus* (46). The findings of the current study showed that early-life nutrition interventions increased the abundance of *Lactobacillus* in the colon. The production and secretion of *MUC2* may have been stimulated by *Lactobacillus* (47). Abundance of *Lactobacillus* was positively correlated with the relative mRNA expression levels of *MUC2*. Furthermore, early-life nutrition interventions downregulated expression of inflammatory cytokines in the present study. This was attributed to increased expression of *MUC2*, and decreased enteric leakiness which in turn reduced contact of intestinal epithelial cells with harmful substances, and ultimately decreased frequency of inflammation (48). Previous human and animal studies indicated that LPS is a strong inducer of proinflammatory cytokines, and is produced by harmful microorganisms (49). LPS stimulation upregulates expression of *TNF-*α, *MCP-1*, and *IL-10* (50). In the present study, Pearson correlation analysis showed that abundances of *Escherichia-Shigella, Parabacteroides, Actinobacillus*, and *Clostridium-sensu-stricto-6* were positively correlated with expression levels of inflammatory cytokines. The lower inflammatory cytokines of piglets in the ENI group can be attributed to the effects of the early-life nutrition interventions that reduced abundances of proinflammatory bacteria such as *Escherichia-Shigella* (51). Previous studies indicate that intestinal crypt hyperplasia can cause a series of intestinal disorders (52). The crypt depth of the colon was increased by early-life nutritional intervention in the present trial. Although similar results were reported by Greeff et al. (8), the study did not fully explore the mechanism underlying increase in crypt length. Further studies should be conducted to explain this phenomenon.

## Conclusion

In summary, early-life nutrition interventions through administration of synthetic milk increased growth performance, improved gut health, and reduced the diarrhea rate of piglets in the present study. Intestinal microbes, mainly beneficial bacteria such as *Lactobacillus*, play an important role in these changes. Implementation of this approach and further studies can improve pig production through reduction of antimicrobial infections thus improving mortality and morbidity of piglets.

## Data Availability Statement

The datasets presented in this study can be found in online repositories. The names of the repository/repositories and accession number(s) can be found below: https://www.ncbi.nlm.nih.gov; PRJNA779481.

## Ethics Statement

The animal study was reviewed and approved by Experimental Animal Welfare and Ethical Committee of the Institute of Animal Science, Chinese Academy of Agricultural Sciences. Written informed consent was obtained from the owners for the participation of their animals in this study.

## Author Contributions

CL and BX designed the experiment. CL, BX, DS, and JL carried out the experiment. CL, BX, and JL wrote the manuscript. RZ, LC, and HZ revised the manuscript. All authors contributed to the article and approved the submitted version.

## Funding

This research was supported by the Agricultural Science and Technology Innovation Program (CAAS-ZDRW202006-02, ASTIPIAS07) and Central Public-Interest Scientific Institution Basal Research Fund (No. 2021-YWF-ZYSQ-01).

## Conflict of Interest

The authors declare that the research was conducted in the absence of any commercial or financial relationships that could be construed as a potential conflict of interest.

## Publisher's Note

All claims expressed in this article are solely those of the authors and do not necessarily represent those of their affiliated organizations, or those of the publisher, the editors and the reviewers. Any product that may be evaluated in this article, or claim that may be made by its manufacturer, is not guaranteed or endorsed by the publisher.
